# Declaración de posición del Grupo de la Comisión Lancet de Hipertensión con respecto a la mejora mundial de las normas de exactitud para los dispositivos de medición de la presión arterial*

**DOI:** 10.26633/RPSP.2020.21

**Published:** 2020-03-01

**Authors:** James E. Sharman, Eoin O’Brien, Bruce Alpert, Aletta E. Schutte, Christian Delles, Michael Hecht Olsen, Roland Asmar, Neil Atkins, Eduardo Barbosa, David Calhoun, Norm R.C. Campbell, John Chalmers, Ivor Benjamin, Garry Jennings, Stéphane Laurent, Pierre Boutouyrie, Patricio Lopez-Jaramillo, Richard J. McManus, Anastasia S. Mihailidou, Pedro Ordunez, Raj Padwal, Paolo Palatini, Gianfranco Parati, Neil Poulter, Michael K. Rakotz, Clive Rosendorff, Francesca Saladini, Angelo Scuteri, Weimar Sebba Barroso, Myeong-Chan Cho, Ki-Chul Sung, Raymond R. Townsend, Ji-Guang Wang, Tine Willum Hansen, Gregory Wozniak, George Stergiou, en nombre del Grupo de la Comisión Lancet de Hipertensión

**Affiliations:** 1 Menzies Institute for Medical Research Universidad de Tasmania HobartTasmania Australia Menzies Institute for Medical Research, Universidad de Tasmania, Hobart, Tasmania, Australia.; 2 The Conway Institute University College Dublin Dublín Irlanda The Conway Institute, University College Dublin, Dublín, Irlanda.; 3 Centro de Ciencias de la Salud Universidad de Tennessee MemphisTennessee Estados Unidos Centro de Ciencias de la Salud, Universidad de Tennessee, Memphis, Tennessee, Estados Unidos (retirado); 4 Equipo de investigación en hipertensión en África, Medical Research Council Unit for Hypertension and Cardiovascular Disease Universidad del Noroeste Potchefstroom Sudáfrica Equipo de investigación en hipertensión en África, Medical Research Council Unit for Hypertension and Cardiovascular Disease, Universidad del Noroeste, Potchefstroom, Sudáfrica.; 5 Institute of Cardiovascular and Medical Sciences Universidad de Glasgow Glasgow Reino Unido Institute of Cardiovascular and Medical Sciences, Universidad de Glasgow, Glasgow, Reino Unido.; 6 Departamento de Medicina Interna, Hospital Holbaek, Holbaek, Dinamarca; y Centre for Individualized Medicine in Arterial Diseases (CIMA), Hospital de la Universidad de Odense Universidad de Dinamarca Meridional OdenseDinamarca Dinamarca Departamento de Medicina Interna, Hospital Holbaek, Holbaek, Dinamarca; y Centre for Individualized Medicine in Arterial Diseases (CIMA), Hospital de la Universidad de Odense, Universidad de Dinamarca Meridional, Odense, Dinamarca.; 7 Institutos de la Fundación para la Investigación Médica Institutos de la Fundación para la Investigación Médica Ginebra Suiza Institutos de la Fundación para la Investigación Médica, Ginebra, Suiza.; 8 Medaval Ltda Medaval Ltda Dublín Irlanda Medaval Ltda., Dublín, Irlanda.; 9 Liga para la hipertensión de Porto Alegre Liga para la hipertensión de Porto Alegre Porto Alegre Brasil Liga para la hipertensión de Porto Alegre, Porto Alegre, Brasil.; 10 Grupo de biología vascular e hipertensión Universidad de Alabama en Birmingham Birmingham Estados Unidos Grupo de biología vascular e hipertensión, Universidad de Alabama en Birmingham, Birmingham, Estados Unidos.; 11 Departamento de Medicina, Fisiología y Farmacología y Ciencias de la Salud Comunitaria, O’Brien Institute for Public Health y Libin Cardiovascular Institute of Alberta Universidad de Calgary CalgaryAlberta Canadá Departamento de Medicina, Fisiología y Farmacología y Ciencias de la Salud Comunitaria, O’Brien Institute for Public Health y Libin Cardiovascular Institute of Alberta, Universidad de Calgary, Calgary, Alberta, Canadá.; 12 George Institute for Global Health Universidad de Nueva Gales del Sur SídneyNueva Gales del Sur Australia George Institute for Global Health, Universidad de Nueva Gales del Sur, Sídney, Nueva Gales del Sur, Australia.; 13 American Heart Association American Heart Association DallasTexas Estados Unidos American Heart Association, Dallas, Texas, Estados Unidos.; 14 Facultad de Medicina de Sídney Universidad de Sídney y Baker Heart & Diabetes Institute MelbourneVictoria Australia Facultad de Medicina de Sídney, Universidad de Sídney y Baker Heart & Diabetes Institute, Melbourne, Victoria, Australia.; 15 Departamentos de Farmacología, Hospital Europeo Georges Pompidou, Assistance Publique Hôpitaux de Paris Inserm UMR 970 y Universidad Paris Descartes París Francia Departamentos de Farmacología, Hospital Europeo Georges Pompidou, Assistance Publique Hôpitaux de Paris, Inserm UMR 970 y Universidad Paris Descartes, París, Francia.; 16 FOSCAL, Instituto Masira, Facultad de Ciencias de la Salud UDES Bucaramanga Colombia FOSCAL, Instituto Masira, Facultad de Ciencias de la Salud, UDES, Bucaramanga, Colombia.; 17 Nuffield Department of Primary Care Health Sciences Universidad de Oxford, Radcliffe Observatory Quarter Oxford Reino Unido Nuffield Department of Primary Care Health Sciences, Universidad de Oxford, Radcliffe Observatory Quarter, Oxford, Reino Unido.; 18 Laboratorio de Investigación Cardiovascular y Hormonal Departamento de Cardiología del Kolling Institute, Royal North Shore Hospital y Facultad de Medicina y Ciencias de la Salud, Universidad Macquarie SídneyNueva Gales del Sur Australia Laboratorio de Investigación Cardiovascular y Hormonal, Departamento de Cardiología del Kolling Institute, Royal North Shore Hospital y Facultad de Medicina y Ciencias de la Salud, Universidad Macquarie, Sídney, Nueva Gales del Sur, Australia.; 19 Departamento de Enfermedades no Transmisibles y Salud Mental Organización Panamericana de la Salud Washington, D.C Estados Unidos Departamento de Enfermedades no Transmisibles y Salud Mental. Organización Panamericana de la Salud, Washington, D.C., Estados Unidos.; 20 Departamento de Medicina, Universidad de Alberta Departamento de Medicina, Universidad de Alberta EdmontonAlberta Canadá Departamento de Medicina, Universidad de Alberta, Edmonton, Alberta, Canadá.; 21 Studium Patavinum, Universidad de Padua Studium Patavinum, Universidad de Padua Padua Italia Studium Patavinum, Universidad de Padua, Padua, Italia.; 22 Istituto Auxologico Italiano, IRCCS Departamento de Ciencias Cardiovasculares, Neurales y Metabólicas, Hospital San Luca, Milán, Italia; y Departamento de Medicina y Cirugía, Universidad de Milán-Bicocca Milán Italia Istituto Auxologico Italiano, IRCCS, Departamento de Ciencias Cardiovasculares, Neurales y Metabólicas, Hospital San Luca, Milán, Italia; y Departamento de Medicina y Cirugía, Universidad de Milán-Bicocca, Milán, Italia.; 23 Imperial Clinical Trials Unit Imperial College London Londres Reino Unido Imperial Clinical Trials Unit, Imperial College London, Londres, Reino Unido.; 24 Asociación Médica Estadounidense Mejorar los Resultados en Materia de Salud ChicagoIllinois Estados Unidos Asociación Médica Estadounidense, Mejorar los Resultados en Materia de Salud, Chicago, Illinois, Estados Unidos.; 25 Mount Sinai Heart Departamento de Medicina (cardiología), Icahn School of Medicine en Mount Sinai, Nueva York, Estados Unidos, y The James J. Peters VA Medical Center BronxNueva York Estados Unidos Mount Sinai Heart, Departamento de Medicina (cardiología), Icahn School of Medicine en Mount Sinai, Nueva York, Estados Unidos, y The James J. Peters VA Medical Center, Bronx, Nueva York, Estados Unidos.; 26 Departamento de Medicina Universidad de Padua; Unidad de Cardiología, Hospital de Cittadella Padua Italia Departamento de Medicina, Universidad de Padua; Unidad de Cardiología, Hospital de Cittadella, Padua, Italia.; 27 Departamento de Ciencias Médicas, Quirúrgicas y Experimentales Universidad de Sácer Sácer Italia Departamento de Ciencias Médicas, Quirúrgicas y Experimentales, Universidad de Sácer, Sácer, Italia.; 28 Liga para la hipertensión Departamento de Cardiología, Universidad Federal de Goiás Goiânia Brasil Liga para la hipertensión. Departamento de Cardiología, Universidad Federal de Goiás, Goiânia, Brasil.; 29 Departamento de Medicina Interna, Facultad de Medicina de la Universidad Nacional Chungbuk Departamento de Medicina Interna, Facultad de Medicina de la Universidad Nacional Chungbuk Cheongju República de Corea Departamento de Medicina Interna, Facultad de Medicina de la Universidad Nacional Chungbuk, Cheongju, República de Corea.; 30 División de Cardiología Departamento de Medicina Interna, Hospital Kangbuk Samsung, Facultad de Medicina de la Universidad Sungkyunkwan Seúl República de Corea División de Cardiología, Departamento de Medicina Interna, Hospital Kangbuk Samsung, Facultad de Medicina de la Universidad Sungkyunkwan, Seúl, República de Corea.; 31 Facultad de Medicina Perelman Universidad de Pensilvania Filadelfia Estados Unidos Facultad de Medicina Perelman, Universidad de Pensilvania, Filadelfia, Estados Unidos.; 32 Instituto de hipertensión de Shanghai, Hospital Ruijin Facultad de Medicina de la Universidad Shanghai Jiaotong Shanghai China Instituto de hipertensión de Shanghai, Hospital Ruijin, Facultad de Medicina de la Universidad Shanghai Jiaotong, Shanghai, China.; 33 Steno Diabetes Center Copenhagen Steno Diabetes Center Copenhagen Gentofte Dinamarca Steno Diabetes Center Copenhagen, Gentofte, Dinamarca.; 34 Hypertension Center STRIDE-7 Universidad Nacional y Kapodistríaca de Atenas, Facultad de Medicina, Tercer Departamento de Medicina, Hospital Sotiria Atenas Grecia Hypertension Center STRIDE-7, Universidad Nacional y Kapodistríaca de Atenas, Facultad de Medicina, Tercer Departamento de Medicina, Hospital Sotiria, Atenas, Grecia.

**Keywords:** Salud internacional, tecnología biomédica, estándares de referencia, equipos diagnósticos, Biomedical technology, diagnostic equipment, international health, reference standard, Tecnologia biomédica, equipamentos para diagnóstico, saúde global, padrões de referência

## Abstract

La Comisión Lancet de Hipertensión determinó que una medida clave para responder a la carga mundial que representa la hipertensión arterial era mejorar la calidad de las mediciones de la presión arterial, mediante la utilización de dispositivos cuya exactitud haya sido validada. En la actualidad existen 3000 dispositivos comercializados, pero muchos no tienen datos publicados sobre pruebas de exactitud conformes a las normas científicas establecidas. La falta de regulación o su ineficiencia, que permiten la autorización de dispositivos para uso comercial sin una validación oficial, posibilitan este problema. Además, han surgido tecnologías nuevas de medición de la presión arterial (por ejemplo, los sensores sin brazalete) sobre las cuales no existe unanimidad en la comunidad científica con respecto a las normas de exactitud de la medición. En conjunto, estos aspectos contribuyen a la disponibilidad generalizada de tensiómetros de consultorio o domiciliarios que ofrecen una exactitud limitada o incierta, que llevan a diagnósticos, manejo y farmacoterapia inapropiados de la hipertensión a escala mundial. Los problemas más importantes relacionados con la exactitud de los dispositivos de medición de la presión arterial se pueden resolver mediante el requisito regulatorio de una validación independiente obligatoria de los dispositivos, en consonancia con la norma ISO universalmente aceptada. Esta es una recomendación básica y constituye una necesidad internacional acuciante. Otras recomendaciones clave son la elaboración de normas de validación específicas para las tecnologías nuevas de medición de la presión arterial y la publicación en línea de listas de los dispositivos nuevos exactos que están a la disposición de los usuarios y los profesionales de salud. Las recomendaciones están en consonancia con las políticas de la Organización Mundial de la Salud sobre los dispositivos médicos y la atención universal de la salud. El cumplimiento de las recomendaciones aumentará la disponibilidad mundial de dispositivos de medición de la presión arterial que sean exactos y tendrá como efecto un mejor diagnóstico y tratamiento, reduciendo así la carga mundial de la hipertensión.

## Abstract (en)

The Lancet Commission on Hypertension identified that a key action to address the worldwide burden of high blood pressure (BP) was to improve the quality of BP measurements by using BP devices that have been validated for accuracy. Currently, there are over 3 000 commercially available BP devices, but many do not have published data on accuracy testing according to established scientific standards. This problem is enabled through weak or absent regulations that allow clearance of devices for commercial use without formal validation. In addition, new BP technologies have emerged (e.g. cuffless sensors) for which there is no scientific consensus regarding BP measurement accuracy standards. Altogether, these issues contribute to the widespread availability of clinic and home BP devices with limited or uncertain accuracy, leading to inappropriate hypertension diagnosis, management and drug treatment on a global scale. The most significant problems relating to the accuracy of BP devices can be resolved by the regulatory requirement for mandatory independent validation of BP devices according to the universally-accepted International Organization for Standardization Standard. This is a primary recommendation for which there is an urgent international need. Other key recommendations are development of validation standards specifically for new BP technologies and online lists of accurate devices that are accessible to consumers and health professionals. Recommendations are aligned with WHO policies on medical devices and universal healthcare. Adherence to recommendations would increase the global availability of accurate BP devices and result in better diagnosis and treatment of hypertension, thus decreasing the worldwide burden from high BP.

## RESUMO

A Comissão Lancet sobre Hipertensão Arterial identificou que uma iniciativa central para enfrentar a carga mundial da hipertensão arterial seria a melhoria na qualidade da mensuração da pressão arterial pelo uso aparelhos de pressão arterial validados quanto à acurácia. Atualmente, existem mais de 3 000 aparelhos de pressão arterial disponíveis comercialmente; entretanto, muitos não têm dados publicados sobre testes de acurácia realizados de acordo com padrões científicos estabelecidos. Este problema resulta de regulamentação fraca ou inexistente, o que permite a aprovação para uso comercial de dispositivos sem validação formal. Além disso, surgiram novas tecnologias de mensuração da pressão arterial (por exemplo, sensores sem algemas) sem consenso científico quanto aos padrões de acurácia. No conjunto, essas questões contribuem para a oferta generalizada de dispositivos de pressão arterial clínica e domiciliar com acurácia limitada ou incerta, levando a diagnóstico, gerenciamento e tratamento inadequados da hipertensão em escala global. Os problemas mais significativos relacionados com a acurácia dos dispositivos de pressão arterial podem ser resolvidos por regulamentação que imponha a obrigatoriedade de validação independente dos aparelhos de pressão arterial, de acordo com a norma universalmente aceita pela Organização Internacional de Normalização. Esta é uma recomendação fundamental para a qual existe uma necessidade internacional urgente. Outras recomendações essenciais incluem o desenvolvimento de padrões de validação especificamente para novas tecnologias de mensuração da pressão arterial e listas *on-line* de aparelhos com acurácia adequada que sejam acessíveis aos consumidores e profissionais de saúde. As recomendações estão alinhadas com as políticas da Organização Mundial da Saúde (OMS) sobre dispositivos médicos e atenção universal à saúde. A adesão às recomendações aumentaria a oferta global de dispositivos de pressão arterial com acurácia adequada e resultaria em melhor diagnóstico e tratamento da hipertensão arterial, diminuindo assim a carga mundial dessa doença.

La hipertensión es el principal factor de riesgo modificable de las enfermedades cardiovasculares y contribuye a la mayor carga de enfermedad en el mundo (1, 2). Existen deficiencias graves con respecto a la concientización óptima, el diagnóstico y el tratamiento de la hipertensión arterial, problemas que persisten en los países de ingresos bajos, medianos y altos y, en conjunto, ponen de relieve la necesidad de un mejoramiento generalizado a escala poblacional (3). Es tranquilizador el hecho de que si se reconoce adecuadamente la presencia de hipertensión arterial mediante la utilización correcta de dispositivos exactos y protocolos apropiados de medición, se puede reducir de manera considerable el riesgo de aparición de episodios cardiovasculares futuros con medicamentos hipotensores (4) e intervenciones con respecto a la alimentación y el modo de vida (5).

La exactitud en la medición de la presión arterial y el diagnóstico de hipertensión son cruciales porque la clasificación errónea puede acarrear consecuencias médicas graves (6). Una sobreestimación de la presión arterial a partir de una medición inexacta podría llevar a iniciar, y posiblemente continuar durante toda la vida, la administración de medicamentos innecesarios con posibles efectos colaterales, además de los efectos sociales no deseados como la ansiedad, el ausentismo laboral (7), y mayores costos debidos a los seguros médicos y los medicamentos (8). Por el contrario, si la medición inexacta da lugar a una subestimación de la presión arterial, se puede perder una oportunidad de prevenir episodios cardiovasculares evitables. Estos problemas no son insignificantes, pues las inexactitudes sistemáticas relativamente pequeñas pueden generar la clasificación errónea de muchos millones de personas a escala poblacional (9). Por lo tanto, la medición exacta de la presión arterial es considerada uno de los exámenes más importantes en la práctica médica (10). La exactitud de la medición de la presión arterial es un problema grave y frecuente en la práctica clínica contemporánea que se debe tener en cuenta como un aspecto que afecta a la seguridad del paciente.

Una de las medidas clave de la Comisión Lancet de Hipertensión fue mejorar la evaluación de la presión arterial, desde una mejor calidad de las mediciones hasta protocolos aprobados y monitores de presión arterial validados (exactos) (11). Los fabricantes deben ceñirse a procedimientos regulatorios estrictos con el fin de introducir legalmente al mercado un dispositivo para medir la presión arterial. Sin embargo, se han encontrado muchos resquicios legales que favorecen una amplia disponibilidad de instrumentos cuya exactitud se desconoce o es mediocre, con fines de utilización clínica, incluido el autocontrol en el hogar (12-19). Los efectos de los errores desconocidos en la presión arterial influyen en el entorno clínico, los datos epidemiológicos y la investigación, e incluso contribuyen a discrepancias entre las directrices sobre hipertensión (20). De cerca de 3000 dispositivos de presión arterial con brazalete que se encuentran en el mercado actual, menos de 15% tienen evidencia publicada que respalde su exactitud (21). Estos dispositivos no validados se usan en la práctica clínica y su probabilidad de ser inexactos es mayor (22–24). Además, está surgiendo para la venta una diversidad de tecnologías nuevas de medición que utilizan sensores y técnicas sin brazalete, pero existen escasas directrices sobre las normas apropiadas para evaluar su exactitud.

El objetivo del presente documento consiste tanto en resumir la situación actual con respecto a los requisitos regulatorios y las normas de exactitud de los dispositivos de medición de la presión arterial, como en rectificar los problemas mencionados arriba al formular recomendaciones y medidas encaminadas a mejorar la validación y la comunicación de las normas de estos dispositivos. El artículo está en plena consonancia con las políticas de la Organización Mundial de la Salud (OMS) sobre los dispositivos médicos y puede contribuir a catalizar la aplicación integral de las resoluciones conexas de la Asamblea Mundial de la Salud (25, 26). En último término, este trabajo procura facilitar la disponibilidad mundial de dispositivos validados de medición de la presión arterial, aumentar de ese modo la probabilidad de un mejor manejo y disminuir la carga mundial por hipertensión arterial.

## ¿CÓMO SE OBTIENE LA APROBACIÓN DE VENTA DE UN DISPOSITIVO DE PRESIÓN ARTERIAL?

Muchos países cuentan con procedimientos legales vigentes mediante los cuales el fabricante de un dispositivo de medición de la presión arterial tiene que demostrar la conformidad del instrumento con los requisitos regulatorios antes de recibir la aprobación de venta. La explicación detallada de los procedimientos regulatorios específicos en los diferentes países está fuera del ámbito de la presente revisión, pues estos difieren en todo el mundo y comportan un alto grado de complejidad. Sin embargo, los principios generales fundamentales de los procedimientos regulatorios son conseguir que los dispositivos de presión arterial cumplan con normas aceptables de calidad, seguridad, fiabilidad y efectividad (por ejemplo, la idoneidad para los fines previstos).

Si una empresa decide comercializar un dispositivo de medición de la presión arterial debe presentar una solicitud a la autoridad regulatoria competente de la jurisdicción de la venta (por ejemplo, la Administración de Alimentos y Medicamentos de los Estados Unidos; la Dirección de Productos Terapéuticos [Canadá] o la Administración de Productos Terapéuticos [Australia], por mencionar algunas). Cuando la información suministrada por el fabricante satisface los requisitos regulatorios locales, se concede una licencia (o aprobación) de venta del dispositivo en ese país. En general, existen grados progresivos de evaluaciones y requisitos regulatorios a medida que aumenta el nivel del riesgo que puede representar un dispositivo para el usuario. En consecuencia, como los dispositivos de presión arterial se clasifican de riesgo bajo a moderado, los requisitos regulatorios son inferiores a los que rigen los dispositivos médicos de riesgo alto.

Aunque la ley no lo exige, una manera habitual en la que el fabricante de un dispositivo médico demuestra el cumplimiento de los requisitos regulatorios es mostrar que el dispositivo está en conformidad con las normas pertinentes publicadas para ese dispositivo. Las normas son documentos elaborados para definir las características técnicas, los procedimientos y las directrices con el fin de garantizar la calidad, la seguridad y la efectividad de los dispositivos. La Organización Internacional de Normalización (ISO) elabora normas de aplicación mundial, pero también pueden existir normas nacionales o regionales independientes.

## NORMAS PARA EVALUAR LA EXACTITUD DE LOS DISPOSITIVOS DE MEDICIÓN DE LA PRESIÓN ARTERIAL

Las iniciativas de validación de los dispositivos de presión arterial comenzaron oficialmente en los años ochenta (27). En 1987, la Asociación Estadounidense para el Progreso del Instrumental Médico (AAMI) preparó un protocolo clínico de validación de los dispositivos de presión arterial (28, 29), al cual siguió un protocolo equivalente de la Sociedad Británica de Hipertensión (30, 31). En 1999, la Liga Alemana para la Hipertensión preparó otro protocolo (32) y en el 2002 el grupo de trabajo sobre el seguimiento de la presión arterial de la Sociedad Europea de Hipertensión (ESH) elaboró su Protocolo Internacional, que fue revisado en el 2010 (33, 34). En la revisión de estos protocolos se han adoptado criterios más estrictos (29, 31, 34). En el 2004, el Comité Europeo para la Normalización (CEN) publicó otra norma (35) y en el 2009, la ISO elaboró asimismo una norma (36) en gran parte basada en las normas de la AAMI y el CEN. La AAMI adoptó esta norma (37) y en el 2013 se publicó una versión revisada de la norma AAMI/ISO (38).

Si bien los protocolos de validación y las normas ya mencionados tienen semejanzas en cuanto al concepto y los procedimientos básicos, también tienen diferencias metodológicas relacionadas con aspectos importantes como el tamaño muestral requerido, los criterios de selección de los participantes, el procedimiento de validación, la medida de eficacia y los criterios de aprobación (39). Esta variabilidad ha creado confusión en los investigadores, los médicos, los usuarios y los fabricantes sobre el protocolo que debe preferirse y por qué. Además, incluso cuando en principio se siguen los protocolos recomendados, los investigadores pueden hacer afirmaciones incorrectas acerca de la validación (40). Después de tres decenios de esfuerzos persistentes de varias organizaciones prestigiosas para optimizar el proceso de validación, no cabe duda de que la mejor contribución para la ciencia, el público, los órganos de regulación y la industria sería acordar una norma única para la validación de los monitores de la presión arterial con aceptación universal.

En el 2017, los comités AAMI, ESH e ISO pusieron en marcha una iniciativa internacional encaminada a establecer una norma universalmente aceptable para evaluar la exactitud de los dispositivos de medición de la presión arterial (41). Esta norma (ISO 81060-2:2018) (42) unifica la evidencia anterior y reemplazará progresivamente todos los protocolos que se aplicaban antes en todo el mundo. A fin de que esta iniciativa aumente la disponibilidad mundial de dispositivos de presión arterial con exactitud clínica validada, sería necesario que se haga obligatoria la aplicación de la norma ISO reconocida en forma universal con el fin de evaluar la exactitud de los dispositivos de medición de la presión arterial antes de ponerlos en el mercado para que sean usados en los entornos médicos, la comunidad y los hogares.

## PROBLEMAS Y RESULTADOS DE LA VÍA REGULATORIA DE LOS DISPOSITIVOS DE MEDICIÓN DE LA PRESIÓN ARTERIAL

Existen problemas importantes en la transmisión de los datos científicos sobre la exactitud de los dispositivos de presión arterial a los usuarios concretos (15, 16, 41, 43-45). Durante varios decenios se han publicado muchos artículos importantes sobre las deficiencias de la medición de la presión arterial (10, 46), pero la información rara vez se ha difundido más allá de los investigadores y los profesionales de salud que ya conocen los problemas. Los fabricantes pueden conocer las limitaciones del dispositivo con respecto a la exactitud, pero mientras no se vea afectado su mercado de usuarios, existen pocos incentivos para modificar las prácticas.

Un problema fundamental de la regulación es que las autoridades se interesan principalmente en reconocer las características de seguridad de los dispositivos en lugar de su exactitud y eficacia diagnóstica (15, 43). En efecto, los fabricantes no tienen la obligación de verificar la exactitud según normas específicas unificadas ni de divulgar al público los resultados de las pruebas de exactitud. También puede ser difícil hacer cumplir las regulaciones debido a las ambigüedades y el alto grado de complejidad (15). Además, las recomendaciones de las organizaciones de usuarios a menudo se basan en el costo, la facilidad de uso y el aspecto estético en lugar de la exactitud. De manera más insidiosa, algunos fabricantes poco éticos venden en línea dispositivos baratos de medición de la presión para uso domiciliario, con afirmaciones fraudulentas con respecto a la validación (47). Esto aumenta la probabilidad de que los usuarios adquieran dispositivos de precios más bajos sin validación, aunque este es un aspecto que debe confirmarse. Muchos otros problemas, que se resumen en el cuadro 1, han dado lugar a la saturación del mercado con tensiómetros cuya exactitud se desconoce o es cuestionable (21-24).

## MÉTODOS Y TECNOLOGÍAS DE LOS NUEVOS DISPOSITIVOS DE MEDICIÓN DE LA PRESIÓN ARTERIAL

Las directrices clínicas para el diagnóstico y el tratamiento de la hipertensión se han elaborado con base en evidencia obtenida con dispositivos auscultatorios u oscilométricos con brazalete, los cuales siguen siendo la norma recomendada en la práctica médica. Estos métodos se fundamentan en el análisis de señales hemodinámicas en la arteria humeral, que en un principio se pensó que eran una buena estimación de la carga de la presión aórtica central (48), la medida de mayor importancia clínica (49-53). En realidad, los tensiómetros de brazalete varían mucho en cuanto a si sus mediciones representan la tensión aórtica central o incluso la presión humeral y puede existir una amplia variación individual entre la presión arterial sistólica central en la aorta, comparada con la arteria humeral (por ejemplo, una diferencia >30 mmHg) (54). Varios dispositivos disponibles pretenden medir la presión aórtica central, a diferencia de la presión arterial medida con los tensiómetros corrientes de brazalete (55). Sin embargo, aparte de las recomendaciones de las asociaciones profesionales (56), no existe ninguna norma regulatoria para evaluar el desempeño de este tipo de dispositivos.

Hay además un auge de tecnologías nuevas que miden la presión arterial de manera muy diferente a la de los tensiómetros de brazalete. Entre ellas se encuentran diversos tipos de sensores sin brazalete que se ubican en diferentes localizaciones arteriales (por ejemplo, el tórax, la cara, el brazo, la muñeca, el dedo) con monitoreo continuo o “instantáneo” de la presión arterial que usan una variedad de registros directos o indirectos y de métodos de procesamiento de la señal (por ejemplo, oximetría de pulso de reflectancia, sensores de radiofrecuencia, microestructuras que utilizan galgas extensiométricas, sistemas optoelectrónicos, tonometría por aplanamiento, tiempo de tránsito del pulso, aplicaciones para teléfonos inteligentes) durante períodos que pueden prolongarse hasta semanas o meses, y que pueden incluir programas informáticos con funciones de apoyo a la toma de decisiones clínicas (57-66). La utilización de algunos de estos métodos no es sencilla y muchos dependen todavía de la calibración con una medición corriente de la presión arterial efectuada con brazalete; estas características son limitantes. No obstante, este es un terreno en evolución continua que ofrece grandes oportunidades para mejorar la evaluación y el control de la hipertensión y, además, para mejorar los sistemas de prestación de servicios de salud al vincularse con los expedientes médicos electrónicos. Sin embargo, estas diversas tecnologías también plantean nuevos retos regulatorios para una supervisión apropiada con respecto al desempeño clínico, la exactitud, la seguridad y la utilidad (67).

**CUADRO 1.** Resumen de los problemas regulatorios y de los estudios de validación, sus resultados y consecuencias relacionados con la exactitud de los dispositivos de medición de la presión arterial

Problema	Resultado	Consecuencias generales
No es obligatorio que los fabricantes utilicen una norma específica para evaluar la exactitud de los dispositivos de medición de la presión arterial No es obligatorio que las pruebas de validación sean realizadas por instancias independientes Varios estudios de validación publicados se apartan de los protocolos establecidos Varios dispositivos de presión arterial han cumplido los requisitos regulatorios de venta pero no han aprobado una validación independiente respecto de la exactitud de la medición Los dispositivos de presión arterial pueden producir lecturas inexactas en personas con brazos grandes o pequeños pero se utilizan incluso en estos casos El supuesto que un dispositivo de presión arterial "autorizado" por las autoridades regulatorias y comercializado es exacto Los requisitos regulatorios se centran en la seguridad en lugar de la exactitud (desempeño) Es posible que no se publiquen los resultados de los dispositivos de presión arterial que no aprueban los estudios de validación No es claro cuál dispositivo de presión arterial se utilizó en el estudio de validación Empresas poco éticas venden en línea dispositivos de presión arterial destinados al hogar con credenciales de validación falsas Las organizaciones de usuarios no prestan la atención debida a la exactitud de los dispositivos de medición de la presión arterial	Utilización de métodos variables para evaluar e informar sobre la exactitud de los dispositivos de medición de la presión arterial Realización de pruebas internas en las empresas con pericia discutible y conflictos de intereses Resultados discutibles y conclusiones injustificadas de los estudios que evalúan los monitores de la presión arterial Mensajes erróneos con respecto a la exactitud de dispositivos específicos de medición de la presión arterial En las personas con brazos demasiado pequeños o demasiado grandes la evaluación de la presión arterial puede ser inexacta Confusión en cuanto a cuál dispositivo de presión arterial disponible en el mercado ofrece una exactitud aceptable Los dispositivos de presión arterial ofrecen un desempeño satisfactorio con respecto a la seguridad, pero pueden ser inexactos Falta de amplia divulgación y transparencia de los resultados de los estudios de validación Confusión en cuanto a si el dispositivo de presión arterial se ha sometido a pruebas de validación Los usuarios pueden preferir la compra de productos baratos, lo cual puede tener consecuencias específicas en las personas de países de ingresos bajos Se da al público en general un asesoramiento inapropiado sobre los mejores dispositivos de presión arterial existentes	Los dispositivos de presión arterial inexactos se encuentran ampliamente disponibles para la venta y el uso por parte de los médicos y el público en general, quienes desconocen el problema. Se emiten diagnósticos incorrectos y se toman decisiones de tratamiento inapropiadas 3. Se pierde la oportunidad de llevar a cabo prácticas óptimas de atención médica y de aumentar la eficacia de la prevención de las enfermedades cardiovasculares

## RECOMENDACIONES Y MEDIDAS QUE PERMITEN ABORDAR LOS PROBLEMAS RECONOCIDOS

Recomendación 1: Convergencia hacia el requisito regulatorio mundial de una validación independiente obligatoria de los dispositivos de medición de la presión arterial en consonancia con la norma ISO (ISO 81060-2:2018) universalmente aceptada y publicación, de preferencia en una revista científica con arbitraje externo

Según la OMS, las tecnologías sanitarias de gran calidad, como los dispositivos validados de medición de la presión arterial, son imprescindibles para una prestación efectiva de atención de salud universal (25). En efecto, el Modelo de Marco Regulatorio Mundial de la OMS para los Dispositivos Médicos presenta un método progresivo para implementar y garantizar el cumplimiento de los controles regulatorios de los dispositivos médicos (68). Existe convergencia regulatoria cuando los requisitos regulatorios concuerdan entre las diferentes regiones gracias a la adopción de documentos técnicos o mecanismos regulatorios reconocidos internacionalmente en consonancia con el objetivo de alcanzar una meta común de salud pública (69). La norma ISO satisface la necesidad urgente de un protocolo único reconocido en forma universal para la validación de los dispositivos de medición de la presión arterial. La norma ya ha sido adoptada por Estados Unidos y también por muchos otros países; si la norma se convierte en un requisito regulatorio obligatorio a escala mundial, corregirá la mayoría de los problemas de exactitud de estos dispositivos asociados con los marcos vigentes. Para lograr esto deben adoptarse numerosas medidas en el amplio espectro de interesados directos clave que incluye las organizaciones gubernamentales (por ejemplo, la FDA u otros organismos regulatorios), las organizaciones no gubernamentales (por ejemplo, las sociedades de hipertensión), los investigadores y los profesionales de salud, las revistas científicas que publican contenidos relacionados con la presión arterial, los fabricantes de los dispositivos y los usuarios que los adquieren. Las organizaciones no gubernamentales pueden desempeñar diversas funciones importantes. En efecto, se ha demostrado que la aprobación de un protocolo normalizado de validación por parte de una sociedad de hipertensión induce a los fabricantes a proseguir la validación independiente de los dispositivos de presión arterial (39). En el cuadro 2 se presenta un resumen detallado de las medidas necesarias.

Recomendación 2: Formulación de normas específicas para la validación de las nuevas tecnologías de medición de la presión arterial que no pueden ponerse a prueba usando la norma ISO (ISO 81060-2:2018)

La inundación del mercado mundial con nuevas tecnologías que pretenden medir la presión arterial plantea dificultades a las autoridades regulatorias que promueven además la búsqueda de soluciones innovadoras (67). En lugar de repetir los problemas regulatorios que se encontraron en el pasado con los tensiómetros corrientes de brazalete, existe la oportunidad de elaborar normas internacionales que sean específicas de las nuevas tecnologías y que evalúen apropiadamente su exactitud y validez clínica. En el cuadro 2 se indican las medidas necesarias para cumplir esta recomendación. Del mismo modo que la recomendación 1, tanto el gobierno como las organizaciones no gubernamentales deberán emprender acciones encaminadas a elaborar las normas específicas y legislar para su uso obligatorio antes de la aprobación de venta, pero también respaldar, regular y vigilar el cumplimiento de las normas nuevas. Es necesario que los profesionales de salud eviten utilizar las nuevas tecnologías de medición de la presión arterial para tomar decisiones clínicas hasta que se haya demostrado que el método en cuestión mejora la atención médica.

Recomendación 3: Listas en línea acreditadas de los dispositivos de presión arterial con un informe pormenorizado de los resultados publicados en los estudios de validación

Existe una necesidad urgente de definir los medios para informar a la comunidad científica y la comunidad general cuáles dispositivos de presión arterial se han puesto a prueba y han demostrado una exactitud aceptable. Esto puede lograrse mediante listas en línea de los dispositivos, que detallen los resultados de los estudios de validación llevados a cabo según las normas internacionales (43). Las listas deben ser elaboradas y mantenidas por organizaciones con la pericia suficiente para garantizar el rigor científico y que conservan su independencia de los fabricantes de los dispositivos. Este tipo de listas han sido elaboradas por las sociedades científicas nacionales en el Reino Unido (70) y Canadá (71). La Asociación Médica Estadounidense y la Asociación Estadounidense del Corazón (72), además de un grupo internacional de expertos en la medición de la presión arterial (organización STRIDE-BP), también desarrollaron recursos semejantes para uso general que representan iniciativas importantes sin fines de lucro. A medida que se avanza hacia una norma ISO única, reconocida en forma universal, también se debe trabajar en la elaboración de una lista acreditada de dispositivos de presión arterial reconocida en todo el mundo, a fin de unificar la información y lograr el mayor efecto. En el cuadro 2 se resumen las medidas y los interesados directos clave para poner en práctica esta recomendación.

## MEDIDAS DE EJECUCIÓN DEL GRUPO DE LA COMISIÓN LANCET DE HIPERTENSIÓN

Un equipo ejecutivo del Grupo de la Comisión Lancet de Hipertensión está dirigiendo un programa coordinado de actividades encaminadas a aplicar las recomendaciones y las medidas planteadas en el cuadro 2. Este equipo estará orientado por un grupo asesor constituido por miembros invitados con pericia pertinente en aptitudes diversas (por ejemplo, científicas, técnicas, de promoción de la causa, de ciencias aplicadas) y representantes de los sectores organizativos (por ejemplo, el gobierno y las organizaciones no gubernamentales, las sociedades de profesionales) de las diferentes regiones del mundo. El cabildeo frente a los tomadores de decisiones clave a nivel de los ministerios de salud nacionales es fundamental para una transferencia eficaz de los conocimientos. Asimismo, el equipo ejecutivo está dispuesto a aprovechar las oportunidades de ejecución disponibles a través de los programas y los recursos internacionales existentes como el Programa de la OMS sobre las Enfermedades Cardiovasculares y en cada país con los líderes comunitarios locales y las organizaciones pertinentes (por ejemplo, las fundaciones del corazón y accidentes cerebrovasculares). Los resultados de estas actividades se evaluarán y publicarán. Los interesados pueden comunicarse con el autor para correspondencia del presente artículo.

**CUADRO 2.** Recomendaciones y medidas necesarias por parte de los interesados directos clave para mejorar a escala mundial las normas de exactitud de los dispositivos de presión arterial

Recomendaciones	Interesados directos clave que deben ejecutar las medidas	Medidas
1. Convergencia hacia el requisito regulatorio mundial de una validación independiente obligatoria de los dispositivos de medición de la presión arterial en consonancia con la norma ISO (ISO 81060-2:2018) universalmente aceptada y publicación, de preferencia en una revista científica con arbitraje externo	Órganos regulatorios del gobierno (por ejemplo, la Administración de Alimentos y Medicamentos de Estados Unidos, la Base de Datos Europea de Dispositivos Médicos, la Administración Australiana de Productos Terapéuticos)	• Legislar en favor de una validación independiente obligatoria de los dispositivos de presión arterial según la norma ISO antes de su aprobación de venta • Estipular que los estudios de validación de los dispositivos de presión arterial se registren en un repositorio aceptado antes de la aprobación de venta • Regular el cumplimiento de la norma ISO • Vigilar la conformidad con la norma ISO • Iniciativas de la OMS de apoyo a la aplicación de las resoluciones de la Asamblea Mundial de la Salud con el fin de garantizar un uso efectivo de los dispositivos de presión arterial y el fortalecimiento de los sistemas regulatorios^‡^
	Organizaciones no gubernamentales (por ejemplo, las sociedades de hipertensión, los grupos de defensa frente a las enfermedades cardiovasculares)	• Crear repositorios para el registro de los estudios de validación de los dispositivos de presión arterial que se llevan a cabo en todo el mundo (un concepto similar a ClinicalTrials.gov) • Certificar los establecimientos de investigación para la realización de estudios de validación de los dispositivos de presión arterial • Promoción de la causa y cabildeo con el objeto de obtener una legislación en favor de la utilización de la norma ISO • Respaldar el uso de dispositivos de presión arterial que han aprobado la validación según la norma ISO • Educar a los miembros sobre la importancia del uso de dispositivos de presión arterial que cumplan con la norma ISO • Educar a los pacientes sobre la importancia de la exactitud de los dispositivos de presión arterial y el acceso a dispositivos validados
	Investigadores	• Registrar los estudios de validación de dispositivos de presión arterial en un repositorio aceptado • Transferencia de conocimientos sobre la exactitud de los dispositivos de presión arterial para beneficio de toda la comunidad científica, médica y la población general
	Profesionales de salud	• Utilizar en la práctica clínica solo los dispositivos de presión arterial validados • Velar por la exactitud permanente de los dispositivos de presión arterial utilizados en la práctica clínica mediante controles de mantenimiento según las instrucciones del fabricante • Educar a los pacientes a que solo utilicen los dispositivos de presión arterial validados para el autocontrol en el hogar
	Revistas científicas con arbitraje	• Aceptar para publicación solo los artículos donde la presión arterial se midió usando dispositivos que han aprobado la validación según la norma ISO
	Fabricantes	• Evaluar la exactitud de los dispositivos de presión arterial aplicando la norma ISO por parte de investigadores independientes • Publicar los resultados de las pruebas de validación en los portales web de la empresa • Utilizar un identificador único específico para cada dispositivo de presión arterial que puede recibir nombres diferentes de venta según los distribuidores • Proporcionar a los compradores las instrucciones sobre el procedimiento de mantenimiento periódico del dispositivo específico adquirido
	Usuarios	• Comprar solo dispositivos de presión arterial que hayan aprobado la validación • Garantizar la exactitud permanente de los dispositivos de presión arterial mediante el mantenimiento periódico según las instrucciones del fabricante • Solo adquirir dispositivos de presión arterial cuando su exactitud se puede confirmar en las listas en línea acreditadas^*^
2. Formulación de normas específicas para la validación de las nuevas tecnologías de medición de la presión arterial que no pueden ponerse a prueba usando la norma ISO (ISO 81060-2:2019)	Organizaciones no gubernamentales	• Formular normas internacionales específicas para las tecnologías nuevas de medición de la presión arterial que no se pueden evaluar con las normas ISO vigentes • Respaldar el uso de normas internacionales para las tecnologías nuevas de medición de la presión arterial
	Organizaciones gubernamentales	• Legislar en favor de la validación independiente obligatoria de los nuevos dispositivos de presión arterial según la norma ISO (elaborada por organizaciones no gubernamentales) antes de la aprobación de venta • Regular el cumplimiento de las normas internacionales • Vigilar la conformidad con las normas internacionales
	Investigadores e ingenieros biomédicos	• Participar en la formulación de normas internacionales para las tecnologías nuevas de medición de la presión arterial • Desarrollar nuevas tecnologías para medir con exactitud la presión arterial • Realizar investigaciones que permitan determinar la capacidad de mejorar la atención médica que ofrecen las nuevas tecnologías de medición de la presión arterial
	Profesionales de salud	• Evitar el uso de tecnologías nuevas de medición de la presión arterial hasta que no hayan demostrado que mejoran la atención médica
3. Listas en línea acreditadas de los dispositivos de presión arterial con un informe pormenorizado de los resultados publicados en los estudios de validación^*^	Gobierno y organizaciones no gubernamentales	• Respaldar y promover las listas en línea acreditadas de los dispositivos de presión arterial • Elaborar y mantener una lista acreditada de los dispositivos de presión arterial, reconocida en todo el mundo • Promover el acceso a las listas en línea acreditadas de los dispositivos de presión arterial mediante el Programa de Medicamentos Esenciales y Productos de Salud de la Organización Mundial de la Salud^†^ • Facilitar la adquisición de monitores de presión arterial validados (de listas acreditadas) a fin de mejorar la calidad y la asequibilidad, mediante iniciativas como el Fondo Estratégico de la OPS^††^
	Investigadores	• Participar en los grupos de consulta de expertos para supervisar la credibilidad científica de las listas en línea acreditadas de dispositivos de presión arterial

ISO, Organización Internacional de Normalización.

‡ Asamblea Mundial de la Salud, resoluciones WHA60.29 y WHA67.20, se pueden consultar en http://apps.who.int/gb/ebwha/pdf_files/WHASSA_WHA60-Rec1/S/WHASS1_WHA60REC1-sp.pdf yhttp://apps.who.int/gb/ebwha/pdf_files/WHA67-REC1/A67_2014_REC1-sp.pdf

* Las listas en línea vigentes de los dispositivios de medición de la presión arterial y la información sobre la validación se pueden consultar en https://bihsoc.org/bp-monitors/, https://hypertension.ca/hypertension-and-you/managing-hypertension/measuring-blood-pressure/devices/ https://medaval.ie/device-category/blood-pressure-monitors/ y https://stridebp.org/

† Programa de Medicamentos Esenciales y Productos de Salud de la Organización Mundial de la Salud, se puede consultar en https://www.who.int/medicines/about/en/

†† OPS, Fondo Estratégico de la Organización Panamericana de la Salud, se puede encontrar información en https://www.paho.org/hq/index.php?option=com_content&view=article&id=12167:faqs-strategic-fund&Itemid=1694&lang=es

## CONCLUSIONES

La medición de la presión arterial es un examen médico fundamental que se realiza diariamente a muchos millones de personas en todo el mundo, y es necesario que los profesionales de salud junto con los usuarios tengan confianza en la exactitud de los dispositivos de medición que se utilizan para el tamizaje, el diagnóstico y el tratamiento de la hipertensión. Desafortunadamente, debido a un sinnúmero de problemas reconocidos se ha inundado el mercado mundial con dispositivos de presión arterial que son inexactos o cuya exactitud se desconoce. Esta situación puede tener consecuencias graves con respecto a las prácticas óptimas de atención a las personas en materia de control de la presión arterial. Asimismo, el problema puede agravarse por la introducción generalizada de nuevas tecnologías que pretenden medir la presión arterial. Por fortuna, la mayoría de los problemas encontrados se puede resolver mediante el requisito regulatorio de una validación independiente obligatoria de los dispositivos de presión arterial, en conformidad con la norma ISO recientemente formulada. Otras necesidades clave son la formulación de normas específicas para la validación de las tecnologías nuevas y la publicación en línea de listas acreditadas de los dispositivos de presión arterial que sean accesibles a los profesionales de salud y los usuarios. Un equipo ejecutivo del Grupo de la Comisión Lancet de Hipertensión trabaja en la actualidad en la implementación de las recomendaciones formuladas en la presente declaración de posición. El cumplimiento de estas recomendaciones estaría en consonancia con la política de la OMS para lograr un diagnóstico y tratamiento más certeros de la hipertensión y disminuir la carga que representa la hipertensión en todo el mundo.

### Agradecimientos.

El Grupo de la Comisión Lancet de Hipertensión está constituido por: Michael H. Olsen, Sonia Y. Angell, Samira Asma, Pierre Boutouyrie, Dylan Burger, Julio A. Chirinos, Albertino Damasceno, Christian Delles, Anne-Paule Gimenez-Roqueplo, Dagmara Hering, Patricio López-Jaramillo, Fernando Martinez, Vlado Perkovic, Ernst R. Rietzschel, Giuseppe Schillaci, Aletta E. Schutte, Angelo Scuteri, James E. Sharman, Kristian Wachtell, Ji-Guang Wang.

Las siguientes organizaciones e instituciones respaldan la presente declaración de posición: Grupo de la Comisión Lancet de Hipertensión, Asociación Estadounidense del Corazón Asociación Médica Estadounidense, Artery Society, Sociedad Brasileña de Cardiología, Sociedad Británica e Irlandesa de Hipertensión, Liga China para la Hipertensión, Fundación Danesa del Corazón, Sociedad Danesa de Hipertensión, grupo de trabajo sobre la vigilancia de la presión arterial y la variabilidad cardiovascular de la Sociedad Europea de Hipertensión, Sociedad Finlandesa de Hipertensión, Sociedad Francesa de Hipertensión, Consejo de Investigación sobre Hipertensión de Australia, Hypertension Canada, Sociedad Internacional de Salud Vascular, Sociedad Coreana de Hipertensión, Sociedad Latinoamericana de Hipertensión, Fundación Nacional del Corazón de Australia, Sociedad Noruega de Hipertensión, Organización Panamericana de la Salud, Resolve to Save Lives, Sociedad Sueca de Hipertensión, Stroke and Vascular Medicine, Liga Mundial contra la Hipertensión.

### Financiación.

Ninguna.

### Declaración.

Las opiniones expresadas en este manuscrito son responsabilidad del autor y no reflejan necesariamente los criterios ni la política de la RPSP/PAJPH y/o de la Organización Panamericana de la Salud.
